# Human Vascular Smooth Muscle Function and Oxidative Stress Induced by NADPH Oxidase with the Clinical Implications

**DOI:** 10.3390/cells10081947

**Published:** 2021-07-31

**Authors:** Kazumi Takaishi, Hiroyuki Kinoshita, Shingo Kawashima, Shinji Kawahito

**Affiliations:** 1 Department of Dental Anesthesiology, Graduate School of Biomedical Sciences, Tokushima University, 3-18-15, Kuramoto, Tokushima 770-8504, Japan; takaishi.k@tokushima-u.ac.jp (K.T.); kawahito.shinji@tokushima-u.ac.jp (S.K.); 2 Department of Anesthesiology, Graduate School of Biomedical Sciences, Tokushima University, 3-18-15, Kuramoto, Tokushima 770-8504, Japan; 3 Department of Anesthesiology and Intensive Care, School of Medicine, Hamamatsu University, 1-20-1, Handayama, Hamamatsu City 431-3192, Japan; shingokawasima@yahoo.co.jp

**Keywords:** human vascular smooth muscle, NADPH oxidase, oxidase stress

## Abstract

Among reactive oxygen species, superoxide mediates the critical vascular redox signaling, resulting in the regulation of the human cardiovascular system. The reduced form of nicotinamide adenine dinucleotide phosphate oxidase (NADPH oxidase, NOX) is the source of superoxide and relates to the crucial intracellular pathology and physiology of vascular smooth muscle cells, including contraction, proliferation, apoptosis, and inflammatory response. Human vascular smooth muscle cells express NOX1, 2, 4, and 5 in physiological and pathological conditions, and those enzymes play roles in most cardiovascular disorders caused by hypertension, diabetes, inflammation, and arteriosclerosis. Various physiologically active substances, including angiotensin II, stimulate NOX via the cytosolic subunits’ translocation toward the vascular smooth muscle cell membrane. As we have shown, some pathological stimuli such as high glucose augment the enzymatic activity mediated by the phosphatidylinositol 3-kinase-Akt pathway, resulting in the membrane translocation of cytosolic subunits of NOXs. This review highlights and details the roles of human vascular smooth muscle NOXs in the pathophysiology and clinical aspects. The regulation of the enzyme expressed in the vascular smooth muscle cells may lead to the prevention and treatment of human cardiovascular diseases.

## 1. Introduction

Increased intracellular calcium, inducing actin-myosin cross-bridge formation, causes vascular smooth muscle contraction [1,2]. The so-called Ca^2+^-independent mechanisms, including Rho A-Rho kinase, protein kinase C, or mitogen-activated protein kinase signaling, reactive oxygen species, and reorganization of the actin cytoskeleton, also contribute to the augmentation of Ca^2+^ sensitivity in the cells [2]. On the other hand, vascular smooth muscle cells are plastic and demonstrate those phenotypic changes from a contractile to a proliferative state in pathological conditions. Indeed, the increased vascular resistance resulting from the smooth muscle cell proliferation in the blood vessels and the enhanced constriction develop hypertension [2]. Vascular smooth muscle cell dysfunction derived from a metabolic derangement is a significant source of vascular complications in type 2 diabetes mellitus [3]. Therefore, the vascular smooth muscle cell function plays a significant role in developing various cardiovascular diseases.

Oxidative stress is a condition in which the balance between the generation of reactive oxygen species and the antioxidant mechanism is disrupted [4]. The condition enhances diverse intracellular redox signaling pathways following the induction of many cardiovascular pathologies, including arteriosclerosis in human vascular smooth muscle cells, although the studies have been still scarce compared with those concerning the human endothelial function [5]. Of these, reactive oxygen species relate to such pathological conditions, in which superoxide is one of the crucial substances [6], as we will discuss in the following sections. Nicotinamide adenine dinucleotide phosphate oxidases (NADPH oxidase, NOX) are cell membrane-bound pro-oxidant enzymes and produce superoxide and hydrogen peroxide [7]. Therefore, understanding the roles of NOX in vascular smooth muscle cells is increasingly critical to implement preventable and therapeutic interventions toward many human cardiovascular diseases. The information regarding the relationship between vascular pathophysiology induced by NOXs and pharmaceutical formulation used during the perioperative period is also essential for the clinicians involved in perioperative patients’ care.

This article focuses on superoxide’s critical roles and each NOX contributing to oxidative stress in human vascular smooth muscle cells. We summarize findings, including the critical role of superoxide in vascular oxidative stress, expressed variable NOXs, pathophysiology related to oxidative stress induced by NOXs, and the relationship between vascular pathophysiology induced by NOXs and pharmaceutical formulation in the perioperative period regarding the human vascular smooth muscle cells.

## 2. Critical Role of Superoxide in Vascular Oxidative Stress

**Figure 1:**
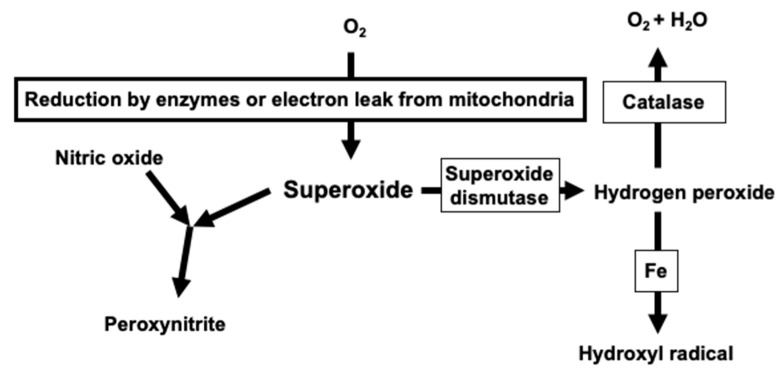
Superoxide and the reactive oxygen species derived from it in human vascular pathology are shown. Superoxide is a critical precursor of reactive oxygen species, including hydrogen peroxide, hydroxyl radical, and peroxynitrite. Superoxide dismutase metabolizes superoxide to hydrogen peroxide, whereas catalase inactivates hydrogen peroxide.

Oxidative stress is when the balance between the generation of reactive oxygen species and the antioxidant mechanism is disrupted [4]. Indeed, superoxide, a precursor of various reactive oxygen species, including hydrogen peroxide, hydroxyl radical, and peroxynitrite, plays a critical role in vascular oxidative stress (Figure 1). The superoxide dismutation catalyzed by an enzyme such as superoxide dismutase produces hydrogen peroxide, resulting in hydroxyl radical via the Fenton reaction and ferrous iron in biological systems [8]. The interaction of superoxide with nitric oxide leads to peroxynitrite production, and thus, causes vascular dysfunction [9]. There are five major pathways known as superoxide production mechanisms in the blood vessels. First, mitochondria produce superoxide as a by-product of oxidative phosphorylation using electrons from the transport chains [10]. Indeed, angiotensin II mediates the human vascular smooth muscle senescence by the mitochondrial superoxide generation, indicating the organelle’s role in human cardiovascular oxidative stress [11]. Second, superoxide can be a co-product of the arachidonic acid metabolism upon the enzymatic activation of cyclooxygenase [12]. A previous study documented that endothelin generates superoxide in a cyclooxygenase-dependent manner, resulting in the impairment of cerebral artery dilation in response to specific K^+^ channels in the experimental animals [12]. Third, the enzymatic activity of xanthine oxidase acting on xanthine is known as a superoxide-generating system, and the system is capable of producing superoxide even in the absence of vascular cells [13]. Xanthine/xanthine oxidase activates mitogen-activated protein kinases, depending on increased levels of extracellular superoxide, resulting in the hypertrophic property in human vascular smooth muscle cells [14]. Fourth, the uncoupled endothelial nitric oxide synthase, resulting from particular oxidative stress, such as the peroxynitrite exposure, or the inhibition of a nitric oxide synthase cofactor tetrahydrobiopterin, produces superoxide in the vascular endothelial cells [15,16]. Fifth, NOX plays a significant role in the superoxide production in vascular structure, as many previous studies documented [17,18]. The single-electron transfer from NADPH to molecular oxygen under the NOX catalysis results in superoxide formation. It is critical to note that NOX is the so-called superoxide (or hydrogen peroxide) production specialist, and therefore, the enzymatic activity is the most predominant source of superoxide in the vascular smooth muscle cells [7].

## 3. NOXs in the Human Vascular Smooth Muscle Cells

Seven different NOX isoforms, including NOX1-5 and DUOX 1/2, have been found in the biological systems, and of these, four subtypes (NOX1, NOX2, NOX4, and NOX5) are known to express in human vascular smooth muscle cells [19,20,21]. It is well known that a NOX isoform, NOX2, was first discovered as a part of the neutrophil bactericidal response, needing massive superoxide production [17,22]. Indeed, neutrophils in chronic granulomatous disease, a group of phagocytic disorders, cannot produce superoxide for host defense [22].

**Figure 2:**
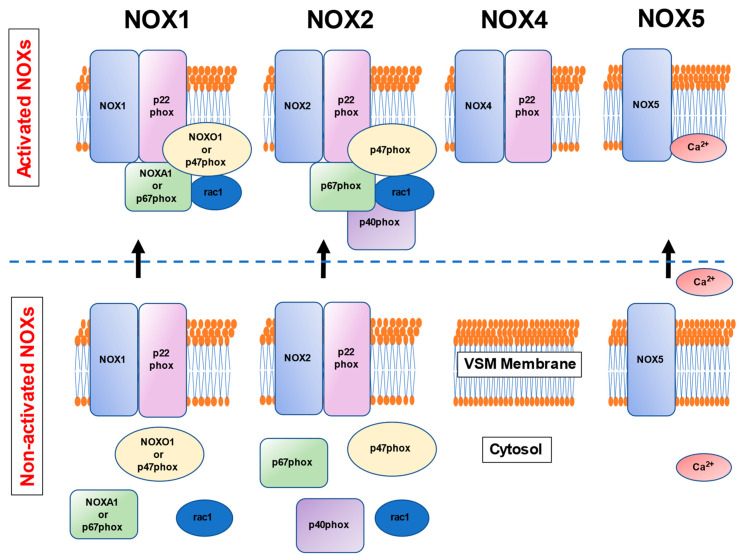
NOX 1, 2, 4, and 5 are expressed in human vascular smooth muscle cells. NOX1, 2, and 4 have the transmembrane subunit p22phox both in the active and inactive forms. NOX1 interacts with the cytosolic subunits NOXO1 or p47phox, NOXA1 or p67phox, and rac1 upon activation. NOX2 activates when it is associated with p47phox, p67phox, p40phox, and rac1. NOX4 is constitutively activated with the cytosolic p22phox subunit. NOX5 needs Ca^2+^ binding in the cytosol for activation.

The human vascular smooth muscle cells-expressed NOX isoforms, NOX1, NOX2, NOX4, and NOX5 isoforms, require an isoform-specific assembly of different subunits or a substance for the activation (Figure 2) [19,20,21]. NOX1 consists of two membrane-associated proteins, p22phox and NOX1, whereas the cytosolic components NOXO1 or p47phox, rac1, and NOXA1 or p67phox translocated to the membrane upon the activation to form the superoxide-generating NOX complex [19,20,21]. NOX2 is comprised of p22phox and NOX2, while p47phox, rac1, p40phox, and p67phox are translocated to the membrane on its activation [19,20,21]. NOX4, which contains p22phox, is distributed in the endoplasmic reticulum and is activated constantly, producing hydrogen peroxide in a predominant fashion [23]. NOX5 requires Ca^2+^ upon its activation [24], leading to increased extracellular vesicles contributing to the cytosolic Ca^2+^ levels in human aortic smooth muscle cells. [25]. However, NOX5 is present in higher mammals, but not in rodents, indicating the difficulty of ascertaining mechanistic insight into human vascular oxidative stress by employing the NOX5-gene knockout mice. A recent study has demonstrated the use of transgenic mice expressing human NOX5 in a vascular smooth muscle cell-specific manner (NOX5 mice) that expresses NOX5 endogenously [26]. In NOX5-expressing mice, agonist-induced vasoconstriction was exaggerated, and a calcium channel inhibitor diltiazem normalized the contractile responses [26]. These results indicate that the NOX5 calcium-dependency may allow pharmacological interventions in humans since many types of calcium antagonists are available in clinical practice.

A recent study has documented that NOX1 and NOX5, but not NOX4, localize in cholesterol-rich fractions in intact human vascular smooth muscle cells from patients undergoing elective craniofacial surgeries [27]. These results suggest an essential role of lipid rafts/caveolae in the signaling platforms for specific NOX isoforms in intact human vascular smooth muscle cells. In other words, the microdomain disruption in cardiovascular diseases probably reduces human vascular smooth muscle oxidative stress resulting from the impairment of a NOX isoform-specific redox signaling.

## 4. Pathophysiology Related to Oxidative Stress Induced by NOX in Human Vascular Smooth Muscle Cells

### 4.1. Hypertension

The increased vascular resistance resulting from smooth muscle cell proliferation in the blood vessels and the enhanced constriction develops hypertension [2]. Vasoactive peptides, including angiotensin II and thromboxane A2, play critical roles in the pathological mechanistic insight into the occurrence and progression of hypertensive disorders in humans [2]. Angiotensin II increases the protein expression of NOX subunits (NOX2, p22phox, p47phox, p40phox, and p67phox) in vascular smooth muscle cells from human resistance arteries derived from gluteal biopsies of healthy subjects [28]. Upon activation by the compound, c-Src regulates the NOX-derived superoxide generation by stimulating p47phox phosphorylation and translocation and chronically by increasing the protein content of NOX2, p22phox, and p47phox in the isolated human vascular smooth muscle cells [29]. The same group further demonstrated that the interaction between p47phox and actin through cortactin plays an essential role in angiotensin II-mediated assembly of functionally active NOX, resulting in reactive oxygen species generation in the vascular smooth muscle cells from human resistance arteries [30]. These findings demonstrate the importance of an actin-cytoskeleton in NOX regulation and redox signaling by angiotensin II in human vascular smooth muscle cells. Angiotensin II induces the phosphorylation of actin-associated protein SM22α, which binds to the actin cytoskeleton, and induces actin bundling to facilitate cytoskeletal structure in a PKCδ pathway-dependent fashion [31]. In human umbilical arterial smooth muscle cells, phosphorylated SM22α activates the protein kinase C-p47phox axis via two pathways, including disassociation of PKCδ from SM22α, and in turn binding to p47phox, in the early stage of angiotensin II stimulation; acceleration of SM22α degradation through ubiquitin-proteasome, enhancing PKCδ membrane translocation via induction of actin cytoskeletal dynamics in later oxidative stress [31]. These studies indicate the link between oxidative stress caused by the p47phox-including NOX1 or NOX2 activation and actin cytoskeletal dynamics upon angiotensin II stimulation toward human vascular smooth muscle cells. On the other hand, another study group documented that angiotensin II enhances the NOX subunit’s mRNA expression of NOX1, NOX4, p67phox, p47phox, and p22phox in cultured human aortic smooth muscle cells, suggesting the possible involvement of NOX1 and NOX4 in the angiotensin II-induced oxidative stress in human vascular smooth muscle cells [32]. A thromboxane A2 analog, U46619, causes increased expression of thromboxane A2 synthase, resulting in augmented thromboxane A2 formation, for which NOX1, but not NOX4, gene-silencing inhibited the synthase activity in human saphenous vein smooth muscle cells from patients undergoing coronary artery bypass graft surgery [33]. The results indicate NOX1 involvement in increased thromboxane A2 synthase expression in human vascular smooth muscle cells.

### 4.2. Hyperglycemia and Diabetes Mellitus

Exposure of the vasculature to metabolic disturbances, including hyperglycemia, adds an impact on vascular walls, specifically on human vascular smooth muscle cells that favor their dysfunction and potentially underlie vascular complications in patients with type 2 diabetes mellitus [3].

We documented that ex vivo incubation with D-glucose (15.5 to 25.5 mmol/L) for 60 min reduces the relaxation and hyperpolarization in response to ATP-sensitive K^+^ channel opener levcromakalim in human omental arteries without endothelium derived from patients without coronary risk factors [6,13,34,35,36]. Superoxide antagonists restored the reduction of vascular smooth muscle function [6,13,34,35,36]. The protein expression of the phosphatidylinositol 3-kinase p85-α subunit and NOX subunits, including p47phox, p22phox, and rac1, increased in these arteries, whereas the Akt phosphorylation at Ser 473, as well as Thr 308 enhanced [6]. A phosphatidylinositol 3-kinase inhibitor LY294002 and superoxide inhibitors restored vasorelaxation and hyperpolarization in response to levcromakalim [6]. Thus, the phosphatidylinositol 3-kinase-Akt pathway activation, combined with the translocation of p47phox, p22phox, and rac1, contributes to the superoxide production induced by high glucose, resulting in the impairment of ATP-sensitive K^+^ channel function in the human visceral artery smooth muscle cells. Takaishi et al. further examined whether the cytoskeleton-disrupting agent reduces oxidative stress caused by D-glucose (25.5 mmol/L) exposed for 60 min in the human omental or coronary artery smooth muscle cells [36]. A cytoskeleton-disrupting agent, cytochalasin B, restored relaxation and hyperpolarization to levcromakalim in human omental arteries exposed to high D-glucose [36]. More importantly, cytochalasin B impaired the F-actin constitution and the membrane translocation of a NOX subunit p47phox, concomitantly reducing superoxide levels in the vascular smooth muscle cells [36]. Collectively, our results indicate that acute exposure for 60 min toward a high D-glucose concentration upregulates NOX1 and NOX2 subtypes, resulting in enhanced oxidative stress in human vascular smooth muscle cells via activation of the phosphatidylinositol 3-kinase-Akt pathway as well as F-actin constitution enhancement.

Levels of NOX-derived superoxide increased in whole internal mammary artery segments from patients with type 2 diabetes mellitus undergoing coronary artery bypass surgery [37]. NOXs activity resulting from p47phox and rac1 membrane translocation enhanced in human internal mammary arteries derived from type 2 diabetes mellitus patients [37]. Guzik et al. also characterized the sources and mechanisms underlying vascular superoxide production in isolated human saphenous veins and internal mammary arteries from diabetic patients with coronary artery disease [38]. NOX’s activity and the levels of NOX protein subunits, including p22phox, p67phox, and p47phox, increased in whole diabetic veins and arteries [38]. These results indicate that enhanced activity of NOX1 and NOX2 contributes to vascular smooth muscle oxidative stress in arteries and veins from patients with type 2 diabetes mellitus.

### 4.3. Inflammation

Tumor necrosis factor-α upregulates NOX activity by enhancing the subunit’s mRNA expression related to NOX1, NOX2, and NOX4 in human aortic smooth muscle cells [32]. Another study by the same group documented that tumor necrosis factor-α increases superoxide production, resulting from the enhanced NOX4 expression in human aortic smooth muscle cells at both message and protein levels, while those of NOX1 and NOX2 were unchanged [39]. The studies suggest a predominant role of NOX4 in tumor necrosis factor-α-induced oxidative stress. A previous study demonstrated that interferon-γ causes increased expression of NOX1 and NOX4 subunits in human aortic smooth muscle cells via the activation of Janus tyrosine kinase/signal transducers and activators of transcription signaling pathway activation, which is an essential pathogenic mechanism leading to smooth muscle cell hypertrophy and hyperplasia [40]. Wu et al. employed mice models with excessive reactive oxygen species production with overexpression of NOX subunit p22phox in vascular smooth muscle cells. They demonstrated that the animals develop high levels of IL-17A and interferon-γ with self-proteins modification producing isoketal-protein adducts [41]. The study indicates a potential mechanism linking oxidative injury caused by p22phox-related NOXs, including NOX1, NOX2, and NOX4, to immune activation and inflammation in mice. More importantly, in human aortas, the aortic content of isoketal adducts correlated with fibrosis and inflammation severity [41]. Therefore, there may be a pathway linking vascular oxidant stress caused by NOXs to immune activation and aortic stiffening in humans. Collectively, NOX4, and possibly NOX1 or NOX2, is likely to be involved in increased superoxide generation in human vascular smooth muscle cells under pro-inflammatory conditions, resulting in variable human vascular pathology.

In contrast, lowering the level of NOX4 below the physiological level leads to cellular senescence of human cultured vascular smooth muscle cells, correlated with secretion of pro-inflammatory cytokines IL-6 and IL-8 [42]. Thus, we have to be cautious when we employ some inhibitors targeting NOX4 toward vascular smooth muscle cells to avoid vascular aging in human vascular diseases.

### 4.4. Arteriosclerosis

Serum derived from patients with chronic kidney disease induces NOX1 upregulation with an elevation of reactive oxygen species and calcium deposition in primary vascular smooth muscle cells [43]. Oxidative stress via NOX1 contributes to vascular calcification in patients with chronic kidney disease, while a NOX1 inhibitor reduced the alteration [43]. These results suggest that reductions in oxidative stress via NOX1 may prevent vascular calcification in patients with chronic kidney diseases.

NOX5 is a unique homolog of NADPH oxidase since it is Ca^2+^-dependent [24]. Vascular calcification, the formation of calcium phosphate crystals in the vessel wall, results in the phenotypic switching from a contractile state to the migrative or proliferative one in vascular smooth muscle cells [25]. Indeed, Ca^2+^-dependent NOX5 increases oxidative stress, which leads to increased extracellular vesicles contributing to the cytosolic Ca^2+^ levels in human vascular smooth muscle cells and subsequent calcification [25]. Guzik et al. noted a marked increase in NOX5 protein and the mRNA levels in isolated coronary arteries from patients with coronary artery disease, but not without the disease [5]. In the study, Ca^2+^-dependent NADPH-driven production of reactive oxygen species in vascular membranes, indicating NOX5 activity, was increased seven-fold in coronary artery disease patients’ specimens, and the activity correlated significantly with NOX5 mRNA levels among subjects [5]. More importantly, the NOX5 protein was expressed in the endothelium in the early lesions and vascular smooth muscle cells in the advanced coronary lesions. Therefore, the Ca^2+^-dependent superoxide-forming NOX5 plays a critical role in arteriosclerosis, and the levels of expression in vascular smooth muscle cells may become a valuable tool for evaluating arteriosclerotic change resulting from oxidative stress in humans.

### 4.5. Heart Failure and Coronary Artery Disease

Guzik et al. examined basal and NOX-mediated superoxide production in isolated human coronary arteries from patients undergoing heart transplantation with and without coronary artery disease [44]. NOX activity and the subunits’ protein levels of p22phox, p67phox, and p47phox in the whole arteries increased in coronary artery disease, and the p22phox and NOX2 mRNA levels enhanced in the vessels [44]. These results indicate the contribution of NOX1 or NOX2 to the increased oxidative stress underlying human coronary artery disease.

Compared with patients with normal left ventricular function, heart failure patients had over two-fold higher superoxide generation and higher expression of the NOX4 and p67phox protein in saphenous vein segments obtained during coronary artery bypass surgery [45]. It is critical to note that superoxide levels were positively correlated with New York Heart Association functional class [45]. Thus, the studies suggest the role of oxidative stress caused by NOX2 and NOX4 in the progression of heart failure.

### 4.6. Marfan Syndrome

Marfan syndrome forms ascending aortic aneurysms, resulting from the altered assembly of extracellular matrix microfibrils and dysfunction of transforming growth factor-β signaling [46]. Jiménez-Altayó et al. identified in aortic aneurysms from Marfan syndrome patients that the activated transforming growth factor-β signaling induces redox stress caused by NOX4 activation [46]. Tyrosine nitration and reactive oxygen species levels with NOX4 expression increased in the tunica media of aortic aneurysms and cultured vascular smooth muscle cells from Marfan syndrome patients [46]. The proteomic analysis further identified nitrated and carbonylated proteins, including smooth muscle α-actin and annexin A2 [46]. These results indicate that the NOX4 isoform impacts the progression of the aortic dilation in Marfan syndrome, the structural organization of the aortic tunica media, and the vascular smooth muscle cells’ phenotypic modulation. However, it is still unclear whether the NOX’s activation is a cause or result in the vascular pathology.

### 4.7. Major Depressive Disorder

Greaney et al. examined the red blood cell flux during graded intradermal microdialysis perfusion in treatment-naïve, otherwise healthy, young adults with major depressive disorder and healthy adults [47]. In the study, both endothelium-dependent and -independent dilations were attenuated in patients with major depressive disorder without sex difference [47]. These results indicate the impairment of vascular smooth muscle dilation in the disease state. In the patients, acute superoxide scavenging improved nitric oxide-dependent dilation, whereas a NOX cytosolic subunit p47phox expression and the activity of oxidative stress markers increased in cutaneous tissues [47]. Therefore, these results suggest that oxidative stress caused by a p47phox-dependent NOX1 or NOX2 impairs the microvascular smooth muscle function in depressive disorder. These findings also support the conclusion that targeting oxidative stress, specifically superoxide, may be a therapeutic strategy to improve vascular function and reduce cardiovascular risk in patients with depression.

### 4.8. Chronic Obstructive Pulmonary Disease

Pulmonary hypertension is an essential negative prognostic sign that develops during the late course of chronic obstructive pulmonary disease [48]. An augmented NOX4 expression is correlated with the increased pulmonary vascular wall volume in lung tissues isolated from patients with chronic obstructive pulmonary disease undergoing lung surgery [49]. In addition, the distal pulmonary artery volume is inversely correlated with pulmonary functions, while it is positively associated with the main pulmonary artery distensibility and right ventricular myocardial mass at both end-systolic and end-diastolic in the disease [49]. The results suggest that the augmented distal pulmonary artery volume indicating pulmonary vascular remodeling reflects the obstructive pulmonary disease severity. More importantly, increased malondialdehyde and decreased levels of superoxide dismutase were seen in serum obtained from chronic obstructive pulmonary disease patients [49]. NOX4-derived reactive oxygen species production may play a role in the pulmonary hypertension development in chronic obstructive pulmonary disease.

### 4.9. Chronic Kidney Disease

Thrombin, platelet-derived growth factor-AB, and transforming growth factor-ß1 upregulated HIF-1α protein in native vascular smooth muscle cells from human renal arteries, and the p22phox antisense oligonucleotides transfection reduced the upregulated protein [50]. These results suggest that a redox-sensitive cascade activated by reactive oxygen species derived from the p22phox-containing NOX is crucially involved in kidney artery disease. Serum derived from patients with early chronic kidney disease stage 2–3 or chronic kidney disease stage 5 induces NOX1 upregulation along with a robust elevation of reactive oxygen species and calcium deposition in primary rat vascular smooth muscle cells [43]. Oxidative stress via NOX1 contributes to vascular calcification in patients with chronic kidney disease, while a NOX1 inhibitor ML171 reduced the alterations [43]. These studies indicate the role of oxidative stress produced by NOX1, NOX2, and NOX4 in the vascular smooth muscle cells of chronic kidney disease patients.

### 4.10. Varicose Veins

A study examined a rac1 inhibitor NSC23766 role in endothelium-dependent vasorelaxation of isolated saphenous vein segments from the patients with and without varicose veins [51]. In the study, the rac1 activity, reactive oxygen species levels, and NOX activity significantly increased in varicose veins, whereas the rac1 inhibitor restored the venous endothelium-dependent relaxation concurrently with reduction of oxidative stress parameter levels in both smooth muscle and endothelium layers [51]. Thus, rac1 pharmacological inhibition resulting in the decreased NOX1 or NOX2 activity may restore human vascular smooth muscle function in a certain cardiovascular disease of humans [6]. Again, it is still unclear whether the NOX’s activation is a cause or result in the vascular pathology.

## 5. Relationship between Vascular Pathophysiology Induced by NOX and Pharmaceutical Formulation Used during the Perioperative Period

### 5.1. Anesthetic Isoflurane

Ex vivo incubation with a high D-glucose concentration (25.5 mmol/L) impairs the relaxation and hyperpolarization induced by an ATP-sensitive K^+^ channel opener levcromakalim in the human omental artery without endothelium [52]. On the contrary, the NOX inhibitor gp91ds-tat and the clinical concentrations of isoflurane (1.15% and 2.3%) restore the relaxation and hyperpolarization in these arteries [52]. Further, isoflurane reduces superoxide production and the intracellular mobilization of a NOX subunit p47phox toward smooth muscle cell membrane in arteries treated with high D-glucose [52]. Therefore, isoflurane preserves ATP-sensitive K^+^ channel activity in human omental artery smooth muscle cells exposed to oxidative stress induced by high D-glucose, whereas the effect appears to be mediated by NOX1 or NOX2 inhibition. These results suggest that a volatile anesthetic protects human visceral arteries from the ion channel malfunction caused by oxidative stress.

### 5.2. Albumin

As we have shown, the incubation with D-glucose (25.5 mmol/L) reduces the levcromakalim-induced dilation of human omental arteries without endothelium, and it increases levels of superoxide and the recruitment of a NOX subunit p47phox in human coronary arterial smooth muscle cells [35]. On the contrary, in the ex vivo model, human serum albumin (0.05 to 0.5 g/dL) prevents the alterations without the superoxide scavenging effect [35]. Interestingly, serum albumin relates to oxidative stress inversely, but the endothelial function positively in pregnant women [35]. Therefore, human serum albumin reduces oxidative stress via NOX1 or NOX2 inhibition in the human vascular smooth muscle cells, and the serum level may be a clinical determinant of vascular oxidative stress in some human diseases.

### 5.3. PPAR Agonist

Regulation of hyperglycemia caused by type 2 diabetes mellitus is essential for the perioperative management in patients undergoing surgery since the pathological state impacts vascular walls, specifically on human vascular smooth muscle cells that favor their dysfunction and potentially underlie vascular complications [3]. The depletion of peroxisome proliferator-activated receptor-γ increases nuclear factor-κB-dependent NOX4 expression and hydrogen peroxide production in human pulmonary artery smooth muscle cells [53]. On the contrary, the NOX4 inhibition attenuated the cell proliferation caused by peroxisome proliferator-activated receptor-γ depletion [53]. Furthermore, human pulmonary artery smooth muscle cells’ peroxisome proliferator-activated receptor-γ overexpression reduces the NOX4 protein expression and cell proliferation [53]. These findings suggest that peroxisome proliferator-activated receptor-γ plays an essential role in regulating the NOX4-hydrogen peroxide signaling axis in human pulmonary artery smooth muscle cells. Thus, the agonists acting on the receptor possibly reduce vascular oxidative stress in humans.

Our previous study documented that peroxisome proliferator-activated receptor-γ agonists, troglitazone and rosiglitazone reduce the superoxide production rate in the ex vivo model using a superoxide-generating system with xanthine-xanthine oxidase in the absence of smooth muscle cells [13]. These findings suggest that synthetic peroxisome proliferator-activated receptor-γ agonists may also play a role as antioxidants.

### 5.4. Vasodilators: Nitric Oxide Donor and Prostacyclin

A nitric oxide donor, NONOate, and a stable prostacyclin analog, iloprost, inhibit superoxide formation, and rac1 and p47phox activation and translocation to the plasma membrane caused by a thromboxane A2 analog U46619 in human saphenous vein smooth muscle cells from patients undergoing coronary artery bypass graft surgery [54]. These findings indicate a possible role of nitric oxide donor and prostacyclin in the oxidative stress reduction via NOX1 or NOX2 inhibition of human vascular smooth muscle cells.

## 6. Summary and Conclusions

Four NOX subtypes (NOX1, NOX2, NOX4, and NOX5) are expressed in human vascular smooth muscle cells [19,20,21]. As described in this review, the above NOXs play critical roles in diverse pathophysiology related to oxidative stress in human vascular smooth muscle cells (Table 1). Vasoactive peptides, including angiotensin II and thromboxane A2, play roles in hypertensive disorders in humans [2]. Previous studies indicate the link between oxidative stress via NOX1, NOX2, and NOX4 caused by these substances involved in hypertension [28,29,30,31,32,33]. NOXs contribute oxidative stress in hyperglycemia and type 2 diabetes mellitus [6,13,34,35,36]. In the ex vivo study, a high D-glucose concentration upregulates NOX1 and NOX2 subtypes [6,13,34,35,36], whereas these NOXs relate to the pathology in vascular smooth muscle cells from patients with type 2 diabetes mellitus similarly [37,38]. Cytokines including, tumor necrosis factor-α, and interferon-γ, upregulate the NOXs’ activity by enhancing the NOX4 (possibly NOX1 and NOX2] expression in the human vascular smooth muscle cells [32,39,40,41]. These results indicate the involvement of NOXs in human vascular smooth muscle cells under pro-inflammatory conditions. Arteriosclerosis is a critical vascular pathology related to many cardiovascular diseases. Serum derived from patients with chronic kidney disease induces NOX1 upregulation with an elevation of reactive oxygen species and calcium deposition in primary vascular smooth muscle cells [43]. Ca^2+^-dependent NOX5 increases oxidative stress, leading to increased cytosolic Ca^2+^ levels in human vascular smooth muscle cells and subsequent calcification [25]. A marked increase in NOX5 protein in isolated coronary arteries from patients with coronary artery disease has been noted [5]. These results suggest that oxidative stress via NOX1 and NOX5 contributes to vascular calcification in human vascular smooth muscle cells. NOX1 and NOX2 activity enhanced in the coronary arteries from patients with coronary artery disease [44], whereas there seems a critical role of oxidative stress caused by NOX2 and NOX4 in the progression of heart failure [45]. Some specific diseases are known to demonstrate oxidative stress caused by NOXs in the vascular smooth muscle cells. The NOX4 isoform impacts the progression of the aortic dilation in Marfan syndrome, the structural organization of the aortic tunica media, and the vascular smooth muscle cells’ phenotypic modulation [46]. Oxidative stress caused by a p47phox-dependent NOX1 or NOX2 seems to impair the microvascular smooth muscle function in depressive disorder [47]. The NOX4-derived reactive oxygen species production is likely to play a role in the pulmonary hypertension development in chronic obstructive pulmonary disease [49]. Previous studies suggest the role of oxidative stress produced by NOX1, NOX2, and NOX4 in the vascular smooth muscle cells of chronic kidney disease patients [43,50]. NOX1 and NOX2 activity increase in varicose veins, concurrently enhancing oxidative stress parameter levels in smooth muscle layers [51]. Whether superoxide or hydrogen peroxide plays a predominant role in the above disease states and whether these reactive oxygen species consistently cause constriction or dilation of human vascular smooth muscle are currently unclear. However, it may be possible that superoxide contributes to somewhat acute vascular pathology, whereas hydrogen peroxide plays a role in the chronic one, as we have shown in part [6,13,15,34,35,52,55].

**Figure 3:**
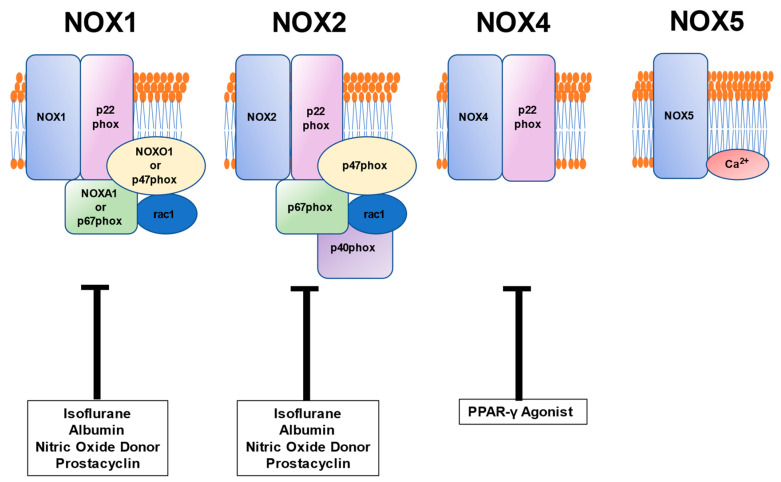
Substances or agents acting on NOXs to reduce the function are shown. Isoflurane, albumin, nitric oxide donor, and prostacyclin possibly inactivate the NOX1 and 2, resulting in decreased oxidative stress in the human vascular smooth muscle cells. In addition, peroxisome proliferator-activated receptor (PPAR)-γ agonists may reduce human vascular oxidative stress since PPAR-γ regulates the NOX4-hydrogen peroxide signaling axis.

The relationship between vascular pathophysiology induced by NOXs and pharmaceutical formulation used during the perioperative period is critical to note for the clinicians involved in the perioperative patients’ care (Figure 3). Anesthetic isoflurane preserves ATP-sensitive K^+^ channel activity in the human vascular smooth muscle cells exposed to oxidative stress, whereas the effect appears to be mediated by NOX1 or NOX2 inhibition [52]. Human serum albumin (0.05 to 0.5 g/dL) reduces oxidative stress via NOX1 or NOX2 inhibition without the superoxide scavenging effect in the human vascular smooth muscle cells [35]. More importantly, serum albumin level may be a clinical determinant of vascular oxidative stress in some human diseases since the level relates to oxidative stress inversely, but the endothelial function positively in humans [35]. Peroxisome proliferator-activated receptor-γ plays an essential role in regulating the NOX4-hydrogen peroxide signaling axis in human artery smooth muscle cells [53]. Thus, the agonists acting on the receptor possibly reduce vascular oxidative stress in humans. In addition, peroxisome proliferator-activated receptor-γ agonists, troglitazone and rosiglitazone, reduce the superoxide production rate in the absence of smooth muscle cells, suggesting the role of these agents as antioxidants in humans [13]. Vasodilators, including a nitric oxide donor and prostacyclin, appear to reduce the oxidative stress caused by NOX1 or NOX2 in human vascular smooth muscle cells [54]. Unfortunately, there seems not to be enough clinical evidence to support the conclusion that we allow using these substances as antioxidants in clinical practice.

In this review, we tried to summarize findings, including the critical role of superoxide in vascular oxidative stress, expressed variable NOXs, pathophysiology related to oxidative stress induced by NOXs, and the relationship between vascular pathophysiology induced by NOXs and pharmaceutical formulation in the perioperative period regarding the human vascular smooth muscle cells. Studies targeting human vascular smooth muscle function have been scarce compared with those concerning human endothelial function, and whether there are any differences in the functioning of pro-oxidant systems, including NOXs, between human arteries and veins is unclear. Thus, further studies are required to understand the fundamental roles of oxidative stress toward vascular smooth muscle cells in human cardiovascular pathologies. Additionally, the information regarding the relationship between vascular pathophysiology induced by NOXs and pharmaceutical formulation is increasingly critical for clinicians. The regulation of the enzyme expressed in the vascular smooth muscle cells may lead to the prevention and treatment of human cardiovascular diseases.

## Figures and Tables

**Table 1:** Pathology related to oxidative stress induced by NOXs and the human vascular smooth muscle cells’ origin.

Pathology	NOX1	NOX2	NOX4	NOX5	Reference Number
**Hypertension**	Peripheral artery	Peripheral artery			[28,29,30]
	Umbilical artery	Umbilical artery			[31]
	Aorta		Aorta		[32]
**Hyperglycemia**	Omental artery	Omental artery			[6]
	Coronary artery	Coronary artery			[36]
**Diabetes Mellitus**	Mammary artery	Mammary artery			[37,38]
	Saphenous vein				[38]
**Inflammation**	Aorta	Aorta	Aorta		[32,39,40]
**Arteriosclerosis**	Aorta				[43]
				Aorta, Coronary artery	[5,25]
**Heart Failure**		Saphenous vein	Saphenous vein		[45]
**Coronary artery disease**	Coronary artery	Coronary artery			[44]
**Marfan Syndrome**			Aorta		[46]
**Depressive disorder**	Cutaneous vessel	Cutaneous vessel			[47]
**COPD**			Pulmonary artery		[48]
**CKD**	Renal artery	Renal artery	Renal artery		[50]
**Varicose Veins**	Saphenous vein	Saphenous vein			[51]

CKD: Chronic kidney disease; COPD: Chronic obstructive pulmonary disease.

## Data Availability

Authors exclude the statement since this study did not report any data.
